# Outcomes of Wide Resection of Soft-Tissue Sarcoma of the Extremity: A Retrospective Analysis

**DOI:** 10.7759/cureus.27329

**Published:** 2022-07-27

**Authors:** Ajay Sheoran, Abhishek Garg, Umesh Yadav, Zile Singh Kundu, Ravi Sherawat, Mohit Singla, Mudit Nemani, Deepender Wason, Harshdeep Singh Kulaar, Sabuj Baran Singha

**Affiliations:** 1 Orthopaedics, Pandit Bhagwat Dayal Sharma Post Graduate Institute of Medical Sciences, Rohtak, IND; 2 Orthopaedics, Positron Multispecialty Hospital, Rohtak, IND; 3 Orthopaedics, General Hospital, Dadri, IND

**Keywords:** soft-tissue sarcoma, recurrence, survival rate, excision, extremity

## Abstract

Background

Soft-tissue sarcomas (STSs) are a rare heterogeneous group of tumors. Good functional results can be achieved with tumor excision in combination with suitable supplemental adjuvant therapies if needed. This study aimed to investigate the outcomes of wide resection of STS of the extremities.

Methodology

In this retrospective study, a total of 139 patients diagnosed with STS of the extremities by radiological and/or histopathological study/biopsy were included. All patients irrespective of metastasis were included.

Results

The mean age of the patients in our study was 43.5 ± 18.89 years. Overall, the mean tumor size was found to be 11.81 ± 6.218 cm. Malignant fibrous histiocytoma was the most common histology encountered (41%). Synovial sarcoma was the second most common histology encountered (14.38%). Recurrence was observed in 14 (10.1%) patients. The overall survival was 64.7% in our study.

Conclusions

The surgical margin achieved during the surgery is the most detrimental factor in local tumor control, and the overall survival of the patient after resection mainly depends on the stage of the tumor.

## Introduction

Soft-tissue sarcomas (STSs) are a group of tumors that account for less than 1% of all adult malignancies. These are malignant mesenchymal neoplasms that share a common embryological and connective tissue origin. Overall, 60% of STSs occur in the extremities. Other locations include the trunk (19%), retroperitoneum (15%), and the head and neck (9%) [1]. Epidemiologically, these tumors have an age-dependent incidence that peaks at over 50 years, although 10-15% of cases can present in children and teenagers [2]. Limb-sparing surgery is the mainstay of surgical treatment for STSs of the extremities [3,4]. In high-grade tumors, micronodules and tumor extensions through the reactive zone around the tumor pseudocapsule can lead to satellite and skip lesions [5]. The resection should be performed outside this reactive zone to avoid recurrence post-surgery. The overall survival varies between 56% and 95%, and disease-free survival ranges from 51% to 92% [6]. This study aimed to evaluate the outcomes of wide resection of STS of the extremities in view of functional outcomes, oncological results (recurrence, metastasis, survival), and complications.

## Materials and methods

This retrospective study was conducted in the Department of Orthopaedics and Pathology from 2012 to 2021 at Pandit Bhagwat Dayal Sharma Post Graduate Institute of Medical Sciences, Rohtak. A total of 139 patients diagnosed with STS of the extremities by radiological and/or histopathological study/biopsy were included in this study. All patients irrespective of metastasis were included. Informed consent was obtained from all patients before enrolling in the study. After confirmation of STS by biopsy/histology, wide resection was performed with a safe margin. The data were retrieved from records of all patients with STS who reported to our Orthopaedic Oncology Clinic and registers maintained in the oncology clinic, histopathological/cytological forms, and registers maintained in the Department of Pathology for the last nine years. The collected data were analyzed in view of the demographic profile including various variables such as name, address, age/sex, location of lesion, size, type of lesion (STS), immunohistochemistry and final histopathological diagnosis, and the kind of surgery performed. Patients were followed up for clinical examination and required investigations. The functional results were evaluated using the Musculoskeletal Tumor Society (MSTS) score on a score of 0 to 5 for upper and lower extremities, including the criteria of pain, function, emotional common to both while hand positioning, manual dexterity, lifting ability for upper limb and supports, walking and gait for the lower limb. The oncological results were evaluated concerning the local recurrence, metastasis, survival, and death. The data were compared with reported literature and the differences, if any, were observed, analyzed, and discussed.

Statistical analysis

Descriptive statistics were analyzed using SPSS version 17.0 software (SPSS Inc., Chicago, IL, USA). Continuous variables were presented as mean ± standard deviation (SD). Categorical variables were expressed as frequencies and percentages. The Pearson’s chi-square test or the chi-square test of association was used to determine the relationship between two categorical variables. A p-value of <0.05 was considered statistically significant. Survival rates were calculated using the Kaplan-Meier method.

## Results

The mean age in our study was 43.5 ± 18.89 years ranging from eight to 86 years. Peak incidence was seen in the fifth decade of life, with the maximum number of cases seen in the age group of 50-60 years (n = 28). There were 83 male (59.7%) patients and 56 female (40.3%) patients in our study. The lower limb was more commonly affected than the upper limb. In 106 (76.26%) cases, the lesion was located in the lower limb, while in 33 (23.74%) cases, the lesion was located in the upper limb. Table 1 demonstrates the distribution of STS in the present study group. The thigh was the most commonly involved site (n = 48), followed by the leg (n = 22). In total, 74 (53.2%) cases presented on the right side of the body and 65 (46.8%) cases on the left half of the body. Hence, the right side was more commonly involved than the right side in our study. Overall, the mean tumor size was found to be 11.81 ± 6.218 cm ranging from 3 to 29 cm.

**Table 1:** Distribution of cases based on the site involved.

	Frequency	Percent (%)
Ankle	4	2.9
Arm	16	11.5
Elbow	2	1.4
Foot	4	2.9
Forearm	11	7.9
Gluteal	9	6.5
Hand	1	0.7
Inguinal	2	1.4
Knee	15	10.8
Leg	22	15.8
Pelvis	1	0.7
Sacrum	1	0.7
Scapula	2	1.4
Shoulder	1	0.7
Thigh	48	34.5
Total	139	100

Table 2 shows the distribution of cases according to the histological type noted in our study. Malignant fibrous histiocytoma was the most common histology encountered (41%). Synovial sarcoma was the second most common histology encountered (14.38%).

**Table 2:** Types of soft-tissue tumors.

Types of soft-tissue tumor	Number of cases	Percent
Malignant fibrous histiocytoma	57	41
Synovial sarcoma	20	14.38
Liposarcoma	19	13.66
Fibrosarcoma	10	7.19
Spindle cell sarcoma	8	5.75
Malignant peripheral nerve sheath tumor	6	4.31
Leiomyosarcoma	5	3.59
Rhabdomyosarcoma	5	3.59
Malignant schwannoma	2	1.43
Undifferentiated sarcoma	2	1.43
Myxoid sarcoma	2	1.43
Epitheloid sarcoma	1	0.71
Myxofibrosarcoma	1	0.71
Total	139	100

Complications

Wound complications (n = 31, 22.3%) and pulmonary metastasis (n = 17,12.2%) were two main complications postoperatively. However, the majority of patients (n = 91, 65.4%) remained asymptomatic after the surgery. Wound complications included wound dehiscence, cellulitis, abscess, seroma, hematoma, and wound necrosis and were mainly noted in the lower extremity, especially in the proximal compartment of the thigh (n = 19, 61.2%). The larger the tumor size the higher the risk of wound dehiscence. The major wound complication rates were 10.7% (7/65), 29.5% (18/61), and 38.4(5/13) for less than 10 cm, greater or equal than 10 cm and smaller than 20 cm, or greater or equal than 20 cm groups, respectively.

Recurrence

In this study, recurrence was observed in 14 (10.1%) patients. The mean interval between the initial surgery and the first recurrence was 37.03 ± 25.16 months (range = 9-71 months). The maximum recurrence that occurred after the initial surgery was within the first two years and was generally identified on routine follow-up. The duration of recurrence according to year is shown in Table 3.

**Table 3:** Duration of recurrence according to year.

Recurrence in year	Frequency	Percentage
<2	7	50
2-3	1	7.1
3-4	2	14.25
>5	4	28.5
Total	14	100%

Functional outcomes

**Figure 1:**
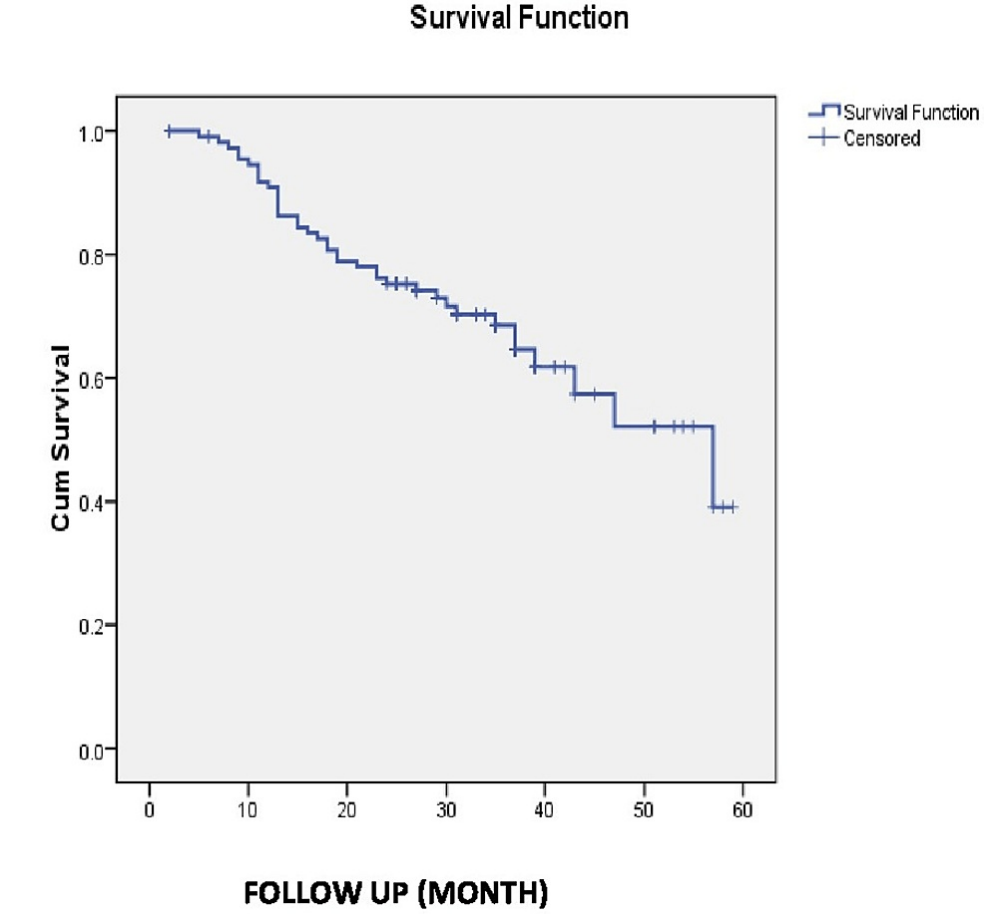
Kaplan-Meier graph showing the five-year survival rate.

In total, 90 (64.7%) patients survived out of 139 over a follow-up of 84 months. Forty-nine (35.3%) patients were dead on follow-up. The overall survival was 64.7% in our study. As shown in Table 4 and Figure 1, the median overall survival follow-up after 84 months was 65 months with a standard error (SE) of 2.58 (95% confidence interval (CI) = 59.943-70.057 months). The mean overall survival after 84 months of follow-up was 57.513 months with an SE of 2.76 (95% CI = 52.104-62.922) and five-year survival was 65.8 months.

**Table 4:** Overall survival statistics.

Mean (months)				Median (months)			
Mean	Standard error	95% confidence interval		Median	Standard error	95% confidence interval	
		Lower	Upper			Lower	Upper
57.513	2.76	52.104	62.922	65	2.58	59.943	70.057

**Figure 2:**
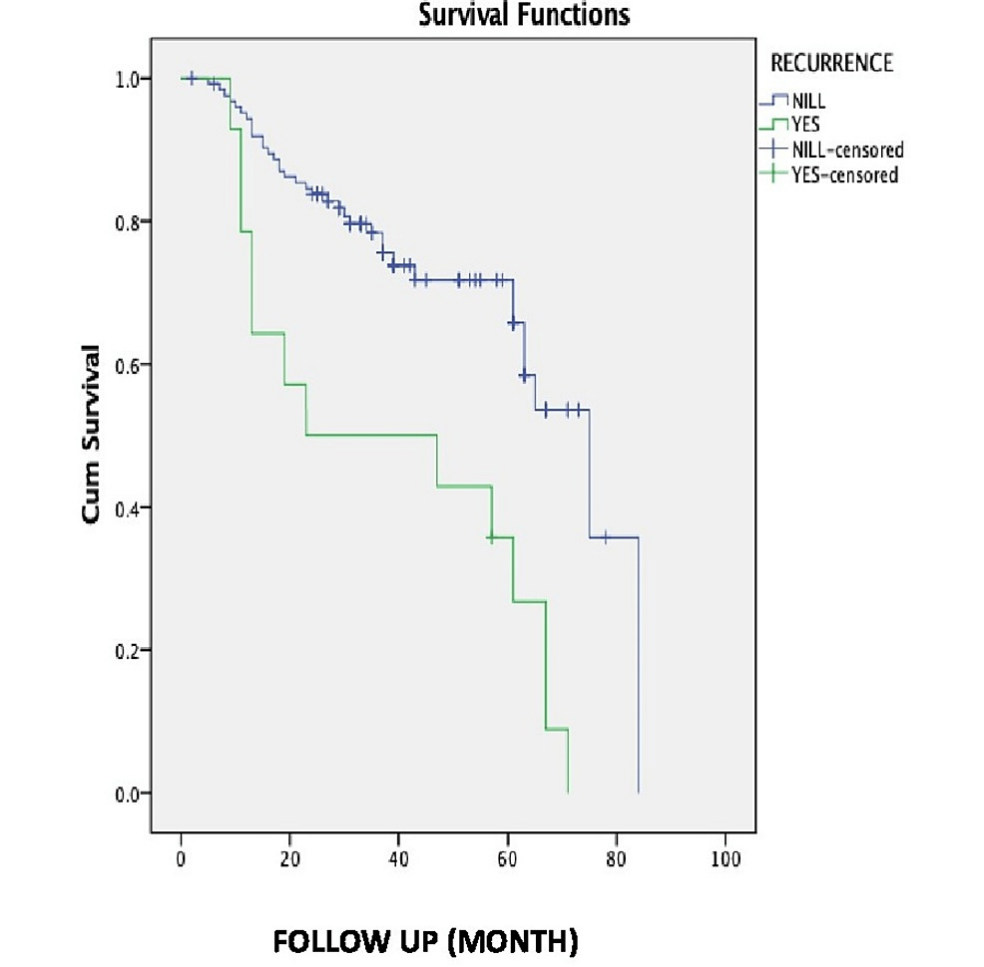
Kaplan-Meier 10-year survival after considering recurrence as a factor.

As shown in Table 5, the mean survival in patients without recurrence was 61.406 months with an SE of 3.097 (95% CI = 55.336-67.475). In the same group, the median survival was 75 months with an SE of 8.209 (95% CI = 58.911-91.089). The mean survival in patients with recurrence was found to be 38.25 months with an SE of 6.941 (95% CI = 24.645-51.855) and the median survival was 23 months (Figure 2).

**Table 5:** Kaplan-Meier statistics taking recurrence as a factor showing the mean/median survival time.

Recurrence	Mean (months)				Median (months)			
	Mean	Standard error	95% confidence interval		Median	Standard error	95% confidence interval	
			Lower	Upper			Lower	Upper
No	61.406	3.097	55.336	67.475	75	8.209	58.911	91.089
Yes	38.25	6.941	24.645	51.855	23	26.192	0	74.336
Overall	57.513	2.76	52.104	62.922	65	2.58	59.943	70.057

**Figure 3:**
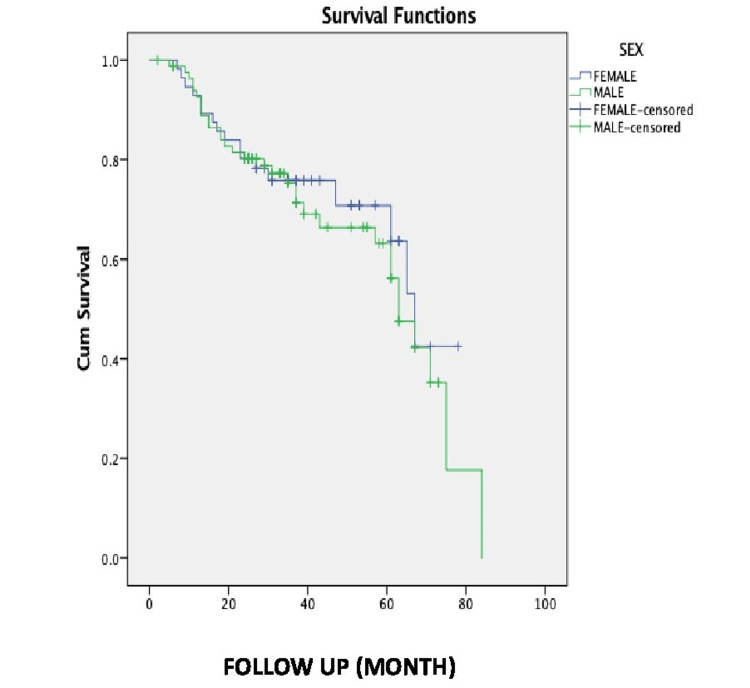
Kaplan-Meier 10-year survival among males and females.

The distribution of mean survival time among genders is shown in Table 6 and Figure 3.

**Table 6:** Distribution of survival time (mean/median) among males and females.

Gender	Mean (months)				Median (months)			
	Mean	Standard error	95% confidence interval		Median	Standard error	95% confidence interval	
			Lower	Upper			Lower	Upper
Male	57.973	3.824	50.478	65.468	67	3.893	59.369	74.631
Female	55.976	3.438	49.238	62.715	63	3.652	55.841	70.159
Overall	57.513	2.76	52.104	62.922	65	2.58	59.943	70.057

The mean survival time in male patients was 57.973 months with an SE of 3.824 (95% CI = 50.478-65.468). The mean survival time in female patients was 55.976 months with an SE of 3.438 (95%CI = 49.238-62.715).

MSTS score

Functional grading performed at the last follow-up (median = 65, mean = 57.5, range = 2-84 months) revealed an overall MSTS score of 20.444 ± 2.35 (range = 16-26 points) out of a possible 30-point total.

## Discussion

STS encompasses wide histological variants such as liposarcoma, fibrosarcoma, and pleomorphic sarcoma. Although these can occur throughout the body, the majority of sarcomas occur in the extremities, with the lower limb being the more preferred site of occurrence [7]. STSs arising in the extremities pose a therapeutic challenge to the treating surgeon due to concerns of functional morbidity and mortality. Function-sparing wide margin surgical excision is the mainstay of the treatment. The goal of the surgery is the preservation of a functional extremity after resection. Wide resection decreases the risk of recurrence but often necessitates complex reconstructions associated with higher rates of surgical complications and increased functional outcomes [1,8].

In this study, the median age was 42 years. Our results are comparable to those of Bajpai et al. [9] (mean age of 41 years) and Dickinson et al. [10] (mean age of 56 years). The lower limb is consistently more commonly involved than the upper limb in various studies in the literature done on different STS [9,11-13]. Our study revealed larger tumor diameters in the lower extremity which corroborates with the findings of Korah et al. [14]. A greater proportion of large-diameter tumors were seen in the proximal lower extremity. Furthermore, tumors located in the adductor compartment were significantly larger in size compared to any other site of the body. Our results confirm the importance of anatomic tumor location. The mean tumor diameter in the present study was found to be 11.81 cm. Similar observations have been reported by Novais et al. [11] who reported a mean tumor size of 11.9 cm in their study. In our study, the maximum number of cases (51.1%) presented with a tumor diameter of more than 10 cm which is consistent with published data, as shown in Table 7 [11,12,15].

**Table 7:** Comparison of various parameters in different studies.

Study	Number of patients	Median age (years)	Mean tumor diameter (cm)	Wound complication rate (%)	Lung metastasis (%)	Overall survival rate (%)
Our study	139	42	11.81	22.3	12.2	57.5
Novais et al. [11]	248		11.9		12.09	65.1
Bajpai et al. [9]	104	41		2.8		64
Potter et al. [15]	363	46			13.8	71
Bansal et al. [7]	43	48		6.9	16.2	47.64
Manoso et al. [6]	42	47		24	10.5	91.3
Gronchi et al. [20]	911	50	6		19.9	

Most histologically diagnosed cases in our study were malignant fibrous histiocytoma (n = 57, 41%). Similar observations were found in various international literature [7,12,16,17]. The histological classification of STSs is an integral part of the diagnostic pathway. Personalized and targeted treatment approaches demand precise sub-classification into one of more than 117 different soft-tissue tumors defined in the recent World Health Organization (WHO) Classification of Bone and Soft Tissue Tumors [18]. Malignant fibrous histiocytoma is the most common histologic type (28%). Others include liposarcoma (15%), leiomyosarcoma (12%), synovial sarcoma (10%), and malignant peripheral nerve sheath tumor (6%) [19].

In our study, the wound complication rate was 22.3% which corroborates with the results of Manoso et al. [6] (24%) and Moore et al. [13] (17.5%). In a study by Bajpai et al. [9], the wound complication rate was 2.8%. In another study by Bansal et al. [7], the postoperative complication rate was 6.9%, as shown in Table 7.

Multiple patient comorbidities have been found to be associated with higher wound complication rates post-surgery. Smoking, obesity, and diabetes have been found to be independent predictors of major wound complications after tumor resection surgery. To reduce postoperative wound complications, more aggressive resection of irradiated soft tissues is required. Moreover, immediate plastic surgery closure should be considered after the surgery, especially if the tumor is located in the adductor compartment of the thigh due to the large size of tumors in this compartment. In our study, the maximum percentage of wound complications was found with a tumor diameter of more than 20 cm. Patients with larger tumors were at a higher risk of wound complications as there was a proportional correlation between tumor diameter and wound complication rate.

Similar results were reported by Moore et al. [13]in their respective study. In our series, out of 139 STS patients, 17 (12.2%) had lung metastasis. The lung metastasis rate of our study is comparable to international studies [7,15,20]. Treatment of pulmonary metastases consisted of a multidisciplinary approach, including wedge resection, chemotherapy, and radiotherapy.

In our study, 14 (10.1%) out of 139 patients developed local recurrences after wide excision of STS. Similar observations were reported by Shukla et al. [21]. Bansal et al. [7] reported a local recurrence rate of 18.6% in their study, while Trovik et al. [16] reported a local recurrence rate of 18% in their study. Livi et al. [22] reported a local recurrence rate of 7.9% in their study. Therefore, the local recurrence of patients with STS in the present study is consistent with international literature, as shown in Table 8.

**Table 8:** Comparison of local recurrence in various studies.

Study	Number of patients	% of local recurrence
Novais et al. [11]	248	28 (11.6%)
Shukla et al. [21]	300	30 (10%)
Trovik et al. [16]	559	101 (18%)
Bansal et al. [7]	43	8 (18.6%)
Livi et al. [22]	214	17 (7.9%)
Potter et al. [15]	363	63 (17.4%)
Present study	139	14 (10.1%)

However, the recurrence rate was slightly lower than in fewer studies, which may be attributed to the relatively smaller sample size of our study. Local recurrence remained an independent predictor of the survival rate after adjusting for all the major risk factors on multivariate analysis. It is generally thought that local recurrence of STS is associated with a poor prognosis and has a negative impact on distant metastasis and the survival of the patient. Novais et al. [11] demonstrated that positive margins increase the risk of local recurrence and diminish the overall survival rate. Similar results of adverse prognostic significance of positive margins on the overall survival of patients were confirmed by Potter et al. [15].

Limb salvage surgery is the mainstay of surgical treatment for STS of the extremities. The benchmark procedure for STS is wide en bloc resection with a reasonable safety margin. However, in high-grade tumors, satellite and skip lesions can be seen due to extensions of the tumor through the reactive zone around the tumor pseudocapsule [12]. To avoid local recurrence after the surgery, tumor resection should be performed outside this reactive zone of the tumor. A 2 cm margin is generally used as a cut-off to ensure adequate tumor clearance after excision. However, at times, the resistant anatomic barriers such as muscular fasciae, periosteum, and perineurium make it difficult to achieve adequate surgical margins during surgery [1,13]. Incomplete resection is one of the significant factors determining local recurrence and overall survival after the surgery. The adequate margin from the tumor for accepting as a negative margin is variable. A study from Helsinki University demonstrated that the surgical margins of >2.5 cm from the tumor were associated with improved local control and better outcomes. They reported that local control rates were 89.2%, 85.9%, and 83.3%, respectively, when combined with adjuvant radiotherapy, with negative margins of at least 2.5 cm, 2 cm, and 1 cm [23].

In our study, the five-year survival rate after wide excision of 139 soft tissue tumors was 65.8%. A similar observation was reported by Novais et al. [11]. The overall survival in our study after 83 months was 57.5%. Moreover, Bajpai et al. reported an overall survival rate of 64% [9]. Potter et al. [15] observed the five-year survival rate and the overall survival rate of 82.6% and 71%, respectively, in their study. While in the study by Manoso et al. [6], the five-year survival rate was 91.3%. Therefore, five-year survival and overall survival are consistent with various international studies in the literature. Smaller tumor sizes and lower histological tumor grades were each independently associated with better functional outcomes after resection of STSs. However, Lee et al. [24] reported that these risk factors did not preclude long-term survival, as 51.2% of patients with tumor size larger than 10 cm and 52.7% of patients with grade 3 tumors were long-term survivors in their study on STS. Although in our study no relationship was studied between the American Society of Anesthesiologists (ASA) score and survival rate, many previous studies [25,26] have suggested a relationship between the ASA score and oncological outcomes of tumors. Lee et al. [24] observed that low ASA scores were significantly associated with long-term survival and better outcomes. This finding suggests that the individual’s general physiological status can independently affect the oncological outcomes after tumor resection. Jacobs et al. [27] reported that the five-year overall survival rates from extremity STSs were significantly improved from 28% to 62% over 20 years. They also observed a slightly increased incidence of extremity STSs over the study period.

In our study, the mean MSTS score was 20.44 ± 2.35. Manoso et al. [6] reported a mean MSTS score of 27.6 in their study. In our study, poor results of MSTS score may trend toward infection and recurrence. Overall, the number of complications, oncological outcomes, and patient survival after surgical wide resection are almost consistent with the reported literature. Our findings, supported by literature, in terms of surgical factors affecting outcome recurrence in STS can be concluded as the following: (a) Smaller tumor size and lower histological tumor grades are associated with better outcomes after resection of STSs. (b) To avoid local recurrence, tumor resection should be performed outside the reactive zone of the tumor. Positive margins increase the risk of local recurrence and diminish the overall survival rate after the resection of STS. (c) The general physiological status of the patient can affect the oncological outcomes after STS resection.

The strength of this study is that all cases were treated in one institution by a single orthopedic oncology surgeon and team. All cases were evaluated histopathologically by pathologists trained in musculoskeletal onco-pathology and the images by the experienced radiologist in collaboration with the operating surgeon. However, we could not exclude other causes of death, and it was not possible to follow each and every patient. Moreover, there were different kinds of lesions with heterogeneous behavior concerning their aggressiveness and tendency to recur/metastasize. Long-term studies are needed to define the exact oncological outcomes after resection of STS. Additional studies are needed to determine the benefit of supplemental adjuvant therapies for systemic tumor control.

## Conclusions

The survival rate drops even after five years post-surgery in high-grade sarcomas. Long-term functional outcomes and the quality of life are not influenced merely by the surgical procedure performed. Good functional results can be achieved by using a combination of tumor resection and additional adjuvant therapies. The surgical margin achieved during the surgery is the most important factor in local tumor control. The overall survival mainly depends on tumor grade. We recommend that STS should be treated in a specialized center with a multidisciplinary setup for optimum treatment outcomes for patients.
